# Trends in mortality among ART‐treated HIV‐infected adults in the Asia‐Pacific region between 1999 and 2017: results from the TREAT Asia HIV Observational Database (TAHOD) and Australian HIV Observational Database (AHOD) of IeDEA Asia‐Pacific

**DOI:** 10.1002/jia2.25219

**Published:** 2019-01-07

**Authors:** In Young Jung, Dhanushi Rupasinghe, Ian Woolley, Catherine C O'Connor, Michelle Giles, Raja ISR Azwa, Jun Yong Choi, IeDEA Asia‐Pacific

**Affiliations:** ^1^ Department of Internal Medicine Yonsei University Wonju College of Medicine Wonju South Korea; ^2^ AIDS Research Institute Yonsei University College of Medicine Seoul South Korea; ^3^ The Kirby Institute UNSW Sydney Sydney NSW Australia; ^4^ Monash Infectious Diseases Monash Health and Monash University Clayton Vic. Australia; ^5^ Department of Infectious Diseases The Alfred Hospital and Monash University Melbourne Vic. Australia; ^6^ Sexual Health Service Sydney Local Health District Camperdown NSW Australia; ^7^ Central Clinical School University of Sydney Sydney NSW Australia; ^8^ University of Malaya Medical Centre (UMMC) Kuala Lumpur Malaysia; ^9^ Department of Internal Medicine Yonsei University College of Medicine Seoul South Korea

**Keywords:** cohort studies, risk factors, mortality, Asia‐Pacific, low‐income, high‐income

## Abstract

**Introduction:**

AIDS‐related deaths in people living with HIV/AIDS have been decreasing in number since the introduction of combination antiretroviral treatment (cART). However, data on recent causes of death in the Asia‐Pacific region are limited. Hence, we analysed and compared AIDS‐related and non‐AIDS–related mortality in high‐ and low‐income settings in the region.

**Methods:**

Patients from the TREAT Asia HIV Observational Database (TAHOD) and Australian HIV Observational Database (AHOD) receiving cART between 1999 and 2017 were included. Causes of death verification were based on review of the standardized Cause of Death (CoDe) form designed by the D:A:D group. Cohorts were grouped as AHOD (all high‐income sites), TAHOD‐high (high/upper‐middle income countries) and TAHOD‐low (lower‐middle income countries). TAHOD sites were split into high/upper‐middle income and lower‐middle income country settings based on World Bank classifications. Competing risk regression was used to analyse factors associated with AIDS and non‐AIDS–related mortality.

**Results:**

Of 10,386 patients, 522 died; 187 from AIDS‐related and 335 from non‐AIDS–related causes. The overall incidence rate of deaths during follow‐up was 0.28 per 100 person‐years (/100 PYS) for AIDS and 0.51/100 PYS for non‐AIDS. Analysis indicated that the incidence rate of non‐AIDS mortality decreased from 0.78/100 PYS to 0.37/100 PYS from year groups 2003 to 2007 to 2013 to 2017 (*p* < 0.001). Similarly, incidence rates of AIDS‐related deaths decreased from 0.51/100 PYS to 0.09/100 PYS from year groups 2003 to 2007 to 2013 to 2017 (*p* < 0.001). More recent years of follow‐up were associated with reduced hazard for non‐AIDS mortality (2008 to 2012: aSHR (adjusted sub‐hazard ratio) 0.72, 95% confidence interval (CI) 0.54 to 0.96, *p* = 0.027; 2013 to 2017: aSHR 0.64, 95% CI 0.47 to 0.87, *p* = 0.004) compared to years 2003 to 2007. The AHOD cohort had almost twice the hazard of non‐AIDS mortality compared to TAHOD‐low (lower‐middle income sites) (aSHR 1.72, 95% CI, 1.20 to 2.46, *p* = 0.003); there were no differences between cohorts for AIDS‐related mortality (*p* = 0.834).

**Conclusion:**

AIDS and non‐AIDS–related mortality rates have decreased over the past years in the Asia‐Pacific region. There is a greater risk for non‐AIDS–associated deaths in the AHOD cohort compared to lower‐middle income settings in TAHOD.

## 1 Introduction

Since the introduction of combination antiretroviral treatment (cART), the incidence and major causes of death among individuals with HIV infection have changed substantially 1, 2, 3, 4. Compared to the pre‐cART era, there has been a decrease in death rates attributable to AIDS itself and an increasing proportion of deaths related to non‐AIDS causes 1, 5. The incidence of AIDS‐defining cancers decreased and the incidence of non‐AIDS‐defining cancers increased after the introduction of cART, due to better viral replication control and increased immunity, which led to longer lifespans and therefore an increased chance of developing comorbidities that are more common with age, such as most non‐AIDS–defining cancers 5. According to the Data collection on Adverse events of anti‐HIV Drugs (D:A:D) study, trends over time in all‐cause mortality in people with HIV demonstrated a decrease in AIDS‐related death rates 1. Moreover, the proportion of non‐AIDS cancers increased from 9% in 1999 to 2000 to 23% in 2009 to 2011 1. A Swiss HIV cohort study demonstrated similar results with 84% of deaths between 2005 and 2009 due to non‐AIDS–related causes 6. Improvement in CD4 cell count was accountable for the recent reductions in rates of AIDS‐related deaths 1.

A previous study conducted in the Asia‐Pacific region examined factors predictive of AIDS‐related and non‐AIDS–related mortality, as well as the cumulative incidences of each cause of death before the year 2007: 215 deaths (89 from AIDS, 97 from non‐AIDS and 29 unknown) were identified, immune deficiency defined by lower CD4 cell counts was predictive of increased risk of AIDS‐related and non‐AIDS–related deaths, and older age (≥50 years) predicted non‐AIDS mortality 7. Other studies in the region have identified risk factors for developing AIDS‐defining cancers 8, 9.

We hypothesize that the causes of AIDS‐related and non‐AIDS–related mortality have changed over the past decade as HIV treatment programs in the Asia‐Pacific region have matured, and that rates of AIDS mortality have decreased, followed by a relative increase in non‐AIDS–related mortality. The objective of this study was to investigate the incidence rates of AIDS‐related and non‐AIDS–related deaths, and identify factors associated with different causes of death (COD) across country income levels in the Asia‐Pacific region during the cART era.

## 2 Methods

### 2.1 Study population and definitions

This analysis includes both Australian HIV Observational Database (Australian HIV Observational Database) and the TREAT Asia HIV Observational Database (TAHOD) enrolled patients who started cART and attended at least one subsequent follow‐up visit. The AHOD and TAHOD are observational clinical cohort studies of patients with HIV infection in Australia and New Zealand (AHOD) and 12 countries (Cambodia, China and Hong Kong SAR, India, Indonesia, Japan, Malaysia, the Philippines, Singapore, South Korea, Taiwan, Thailand, and Vietnam) in Asia and the Pacific region (TAHOD), whose methods have previously been described 7, 8, 9.

The start date (baseline) for cART was defined as the date patients started their first triple regimen. Those who used mono/dual therapy prior to starting cART were excluded. Cohorts were grouped as AHOD (all high‐income sites), TAHOD‐high (high/upper‐middle income countries) and TAHOD‐low (lower‐middle income countries). TAHOD sites were split into high/upper‐middle income and lower‐middle income settings (there are no low‐income sites in TAHOD) based on World Bank classifications 10. Deaths were classified as AIDS‐related where the death was a direct consequence of any AIDS‐defining condition (as defined by the Centers for Disease Control and Prevention 11). Non‐AIDS deaths were further classified as being related to cardiovascular disease, liver disease, cancer, accidental/overdose/violence, infectious disease, other and unknown 1. Causes of death were based on review of the standardized Cause of Death (CoDe) form designed by the D:A:D group 12.

### 2.2 Statistical analysis

#### 2.2.1 Proportions and incidence rates of AIDS‐related and non‐AIDS–related mortality

Proportions of AIDS‐related and non‐AIDS–related mortality were analysed descriptively. Calendar years were categorized into ≤2007, 2008 to 2012 and 2013 to 2017. The denominator used to calculate each proportion was the total number of deaths in each year group. Mortality rates for different causes of deaths were also examined. The proportions and mortality rates were plotted across calendar years of follow‐up. Unknown causes of death were plotted as a separate group to allow for visual inspection of the reported cases of non‐AIDS causes of death.

#### 2.2.2 Factors associated with AIDS‐related and non‐AIDS–related mortality

Fine and Grays’ competing risk regression model was used to analyse factors associated with AIDS‐related and non‐AIDS–related mortality. The resulting sub‐hazard ratio (SHR) can be interpreted in a similar way to the hazard ratio. Follow‐up time began from cART initiation and left truncated at cohort entry for those who entered the cohort after starting cART. Left truncation minimizes survival bias by taking into account survival time prior to cohort entry in those who had initiated cART before enrolment into the cohort. The earliest year of follow‐up started in 1999 for AHOD and 2002 for TAHOD. While follow‐up time ended at date of death for patients who experienced mortality, all other patients were censored on their date of last follow‐up. Both AHOD and TAHOD cohorts had their last follow‐up date in year 2017. Other causes of death and loss to follow‐up (LTFU) 13, 14, 15 were defined as competing events. Time‐fixed covariates were age, sex, HIV mode of exposure, and cohort groups. Patients were considered hepatitis B virus (HBV) or hepatitis C virus (HCV) positive if they were ever tested positive for HBV surface antigen or positive for HCV antibody respectively. Covariates which varied with time were diabetes, hypertension, body mass index (BMI), HIV RNA, CD4 cell count and calendar year (≤2002, 2003 to 2007, 2008 to 2012, and 2013 to 2017). These variables were time‐updated from risk time which began at antiretroviral treatment (ART) initiation or cohort entry for those who had initiated cART prior to cohort entry, till the date of last follow‐up for those who were alive and date of death for those who experienced mortality. Time‐updated variables contribute different risk time in different categories. The missing category in the time‐updated variables refers to missing baseline values or patients who never had a test in the entire follow‐up time. Diabetes was defined as documentation of one fasting blood glucose measurement ≥7 mmol/L. Hypertension was not included as blood pressure was not recorded by the AHOD cohort. BMI was defined as underweight (<18.5), normal range (18.5 to 24.9) and overweight (≥25). LTFU was defined as not having been seen in clinic for >12 months without evidence of transfer.

With the exception of cohort groups, covariates in the univariate analysis with *p* < 0.10 were fitted in the multivariate model using backward stepwise selection process. Covariates with *p* < 0.05 in the multivariate model were considered significant. A sensitivity analysis was performed by including age as a time‐updated covariate, and adjusting for age, sex and CD4 cell count in the final multivariate model.

Ethics approvals for TAHOD were obtained from the respective local ethics committees of all participating sites, the data management and biostatistical centre at the Kirby Institute (The University of New South Wales (UNSW) Human Research Ethics Committee), and the coordinating centre at TREAT Asia/amfAR. Written informed consent was sought in TAHOD if required by a site's local institutional review board. All TAHOD data transfers are anonymized prior to submission to the Kirby Institute. Ethics approval for AHOD was obtained from the respective local ethics committees of all participating sites and the UNSW Human Research Ethics Committee; written informed consent was obtained from all participants. All data management and statistical analyses were performed using SAS software version 9.4 (SAS Institute Inc., Cary, NC, USA) and Stata software version 14.2 (Stata Corp., College Station, TX, USA).

## 3 Results

A total of 10,386 patients who had initiated cART and were in follow‐up between years 1999 and 2017 were included in the study. With a median follow‐up time of 6.0 years, 522 patients (155 from AHOD, 208 from TAHOD‐high and 159 from TAHOD‐low) experienced AIDS or non‐AIDS mortality: 187 died from AIDS‐related (incidence rate of 0.28 per 100 person‐years (PYS)) and 335 died from non‐AIDS–related causes (0.51/100 PYS) (Table 1). In AHOD, the mortality rate for AIDS‐related death was 0.15/100 PYS and for non‐AIDS–related death it was 0.66/100 PYS, with a median follow‐up time of 6.3 years. The mortality rates for TAHOD‐high were 0.29/100 PYS for AIDS and 0.42/100 PYS for non‐AIDS deaths with a median follow‐up of 7.1 years, and for TAHOD‐low the mortality rate was 0.40/100 PYS for AIDS and 0.49/100 PYS for non‐AIDS with a median follow‐up of 5.5 years.

**Table 1:** AIDS and non‐AIDS causes of death in low and high income settings in the TREAT Asia HIV Observational Database and AHOD cohorts^a^

	TAHOD, low income (N)	TAHOD, high income (N)	AHOD, high income (N)	All patients	Incidence rate (/100 PYS) (follow‐up time of 66,250 years)
Total patients in database	3240	4429	2717	10,386	
Causes of death category^b^					
AIDS related	72	86	29	187	0.28
Non‐AIDS related	87	122	126	335	0.51
Cardiovascular disease	10	12	19	41	0.06
Cancer	2	19	37	58	0.09
Liver disease	9	17	14	40	0.06
Accidental/overdose/violence	9	14	11	34	0.05
Infectious disease	11	13	6	30	0.05
Other	13	10	9	32	0.05
Unknown	33	37	30	100	0.15
Total	159	208	155	522	0.79

AHOD, Australian HIV Observational Database; TAHOD, TREAT Asia HIV Observational Database.

^a^Cohorts were grouped as AHOD (all high‐income sites), TAHOD‐high (high/upper‐middle income countries) and TAHOD‐low (low‐middle/low income countries). TAHOD sites were split into high/upper‐middle income and low‐middle/low income settings based on World Bank classifications. ^b^Causes of death were based on review of the standardized Cause of Death (CoDe) form designed by the Data collection on Adverse events of Anti‐HIV Drugs (D:A:D) group.

Of the 522 patients who died, the median age in the TAHOD‐low group was 34 years [interquartile range (IQR) 29 to 40], 41 years [IQR 34 to 51] in the TAHOD‐high group and 42 years [IQR 35 to 53] in AHOD. Ninety‐five per cent of patients in AHOD were men compared with 81% and 75% in TAHOD‐high and TAHOD‐low income groups respectively. Heterosexual contact was the primary route of HIV exposure in TAHOD‐high and TAHOD‐low income sites (67%), whereas men who have sex with men (MSM) was the most common HIV exposure in the AHOD sites (70%). Median baseline CD4 cell counts were 61 cells/μL [IQR 20 to 154] in the TAHOD‐low group, 59 cells/μL [IQR 19 to 164] in the TAHOD‐high group and 210 cells/μL [IQR 87 to 350] in AHOD. Baseline HIV RNA were 190,000 copies/mL [IQR 85,138 to 480,000] in the TAHOD‐low group, 140,000 copies/mL [IQR 47,650 to 450,000] in the TAHOD‐high group and 110,000 copies/mL [IQR 25,228 to 460,000] in AHOD. (Table 2)

**Table 2:** Patient characteristics at baseline in low and high income settings in the TREAT Asia HIV Observational Database and AHOD cohorts^a^

	TAHOD, low income (N)		TAHOD, high income (N)		AHOD, high income (N)		*p*‐value^b^
	n (%)	No. of deaths (%)	n (%)	No. of deaths (%)	n (%)	No. of deaths (%)	
Total	3240 (100)	159 (100)	4429 (100)	208 (100)	2717 (100)	155 (100)	
Age (years)							
Median (IQR)	33 (28 to 38)	34 (29 to 40)	36 (30 to 42)	41 (34 to 51)	38 (31 to 46)	42 (35 to 53)	<0.001
≤30	1213 (37)	55 (35)	1225 (28)	35 (17)	573 (21)	18 (12)	<0.001
31 to 40	1417 (44)	66 (42)	1822 (41)	67 (32)	1000 (37)	54 (35)	
41 to 50	444 (14)	23 (14)	959 (22)	50 (24)	713 (26)	35 (23)	
51+	166 (5)	15 (9)	423 (10)	56 (27)	431 (16)	48 (31)	
Sex							
Male	2172 (67)	120 (75)	3195 (72)	169 (81)	2475 (91)	147 (95)	<0.001
Female	1068 (33)	39 (25)	1234 (28)	39 (19)	242 (9)	8 (5)	
HIV exposure							
Heterosexual	2314 (71)	107 (67)	2522 (57)	139 (67)	530 (20)	18 (12)	<0.001
MSM	235 (7)	9 (6)	1425 (32)	40 (19)	1901 (70)	108 (70)	
Injecting drug use	503 (16)	31 (19)	109 (2)	12 (6)	152 (6)	18 (12)	
Blood	15 (1)	1 (1)	62 (1)	5 (92)	16 (1)	2 (1)	
Bisexual	79 (2)	6 (4)	61 (1)	4 (2)	0 (0)	0 (0)	
Other/unknown	94 (3)	5 (3)	250 (6)	8 (4)	118 (4)	9 (6)	
CD4 cell count (cells/μL)							
Median (IQR)	124 (42 to 225)	61 (20 to 154)	133 (40 to 232)	59 (19 to 164)	304 (180 to 460)	210 (87 to 350)	<0.001
≤100	1199 (37)	80 (50)	1575 (36)	119 (57)	295 (11)	35 (23)	<0.001
101 to 200	691 (21)	32 (20)	893 (20)	30 (14)	324 (12)	24 (15)	
201 to 350	655 (20)	12 (8)	931 (21)	22 (11)	646 (24)	32 (21)	
351 to 500	104 (3)	1 (1)	201 (5)	10 (5)	440 (16)	16 (10)	
501+	75 (2)	1 (1)	92 (2)	1 (1)	430 (16)	13 (8)	
Missing	516 (16)	33 (21)	737 (17)	26 (13)	582 (21)	35 (23)	
Viral load (copies/mL)							
Median (IQR)	130,000 (33,500 to 390,000)	190,000 (85,138 to 480,000)	89,571 (26,650 to 250,000)	140,000 (47,650 to 450,000)	64,702 (14,362 to 180,000)	110,000 (25,228 to 460,000)	<0.001
≤400	28 (1)	0 (0)	120 (3)	5 (2)	190 (7)	7 (5)	<0.001
401 to 10,000	77 (2)	1 (1)	278 (6)	4 (2)	258 (10)	15 (10)	
10,001 to 100,000	209 (6)	5 (3)	1158 (26)	49 (24)	926 (34)	31 (20)	
100,001+	392 (12)	16 (10)	1348 (30)	74 (36)	698 (26)	54 (35)	
Missing	2534 (78)	137 (86)	1525 (34)	76 (37)	645 (24)	48 (31)	
HBV co‐infection surface antigen							
Negative	1984 (61)	91 (57)	3317 (75)	137 (66)	2156 (79)	122 (79)	<0.001
Positive	231 (7)	11 (7)	379 (9)	29 (14)	95 (4)	10 (6)	
Missing	1025 (32)	57 (36)	733 (17)	42 (20)	466 (17)	23 (15)	
HCV co‐infection antibody							
Negative	1439 (44)	57 (36)	3269 (74)	136 (65)	2184 (80)	118 (76)	<0.001
Positive	556 (17)	28 (180	307 (7)	38 (18)	247 (9)	25 (16)	
Missing	1245 (38)	74 (47)	853 (19)	34 (16)	286 (11)	12 (8)	
Diabetes							
No	1121 (34)	48 (30)	1162 (26)	41 (20)	356 (13)	6 (4)	<0.001
Yes	67 (2)	5 (3)	29 (1)	1 (1)	19 (1)	2 (1)	
Missing	2052 (63)	106 (67)	3238 (73)	166 (80)	2717 (86)	147 (95)	
BMI groups							
Underweight (<18.5)	699 (22)	57 (36)	499 (11)	31 (15)	16 (1)	0 (0)	<0.001
Normal range (18.5 to 24.9)	1296 (40)	41 (26)	1590 (36)	74 (36)	221 (8)	9 (6)	
Overweight (≥25)	243 (8)	6 (4)	316 (7)	11 (5)	105 (4)	4 (3)	
Missing	1002 (31)	55 (35)	2024 (46)	92 (44)	2375 (87)	142 (92)	
Prior AIDS							
No	1902 (59)	49 (31)	2930 (66)	90 (43)	2405 (89)	122 (79)	<0.001
Yes	1338 (41)	110 (69)	1499 (34)	118 (57)	312 (11)	33 (21)	
Total lost to follow‐up	532 (2.98/100 PYS)		729 (2.49/100 PYS)		853 (4.45/100 PYS)		

AHOD, Australian HIV Observational Database; BMI, body mass index; HBV, hepatitis B virus; HCV, hepatitis C virus; IQR, interquartile range; MSM, men who have sex with men; No., number; PYS, person‐years; TAHOD, TREAT Asia HIV Observational Database.

^a^Cohorts were grouped as AHOD (all high‐income sites), TAHOD‐high (high/upper‐middle income countries) and TAHOD‐low (lower‐middle income countries). TAHOD sites were split into high/upper‐middle income and lower‐middle income settings based on World Bank classifications. ^b^ *p*‐values are tests for differences in proportions (chi‐squared) or medians (Kruskal‐Wallis test) for total patients across different cohorts. Baseline was defined as the date patients started their first triple regimen.

**Figure 1:**
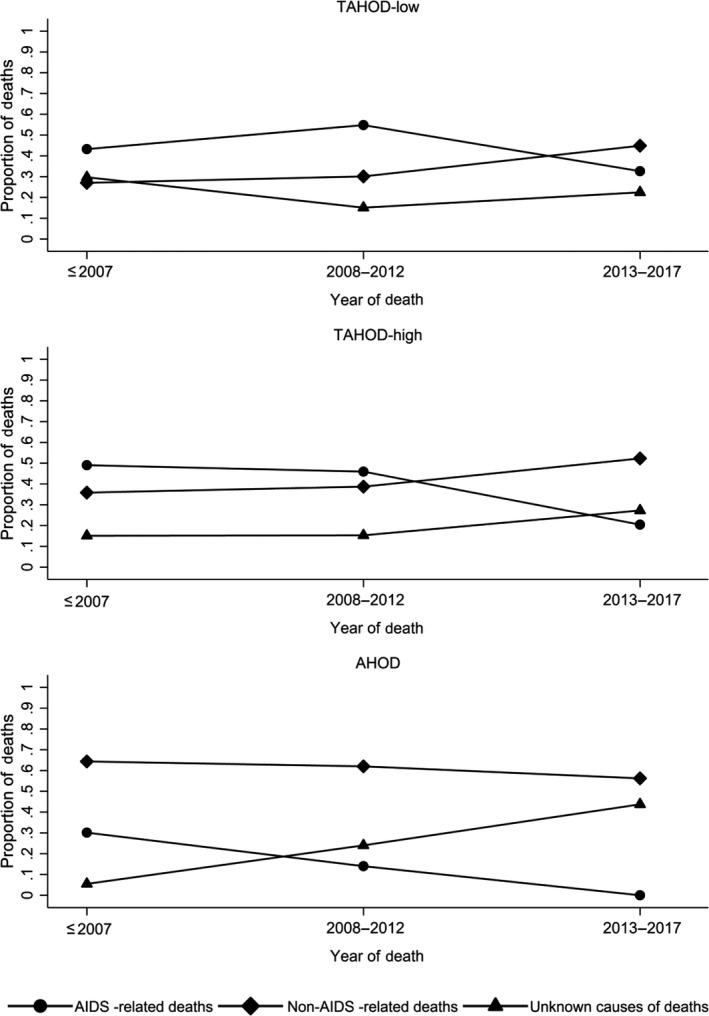
Proportions of AIDS and non‐AIDS mortality among those who have died

**Figure 2:**
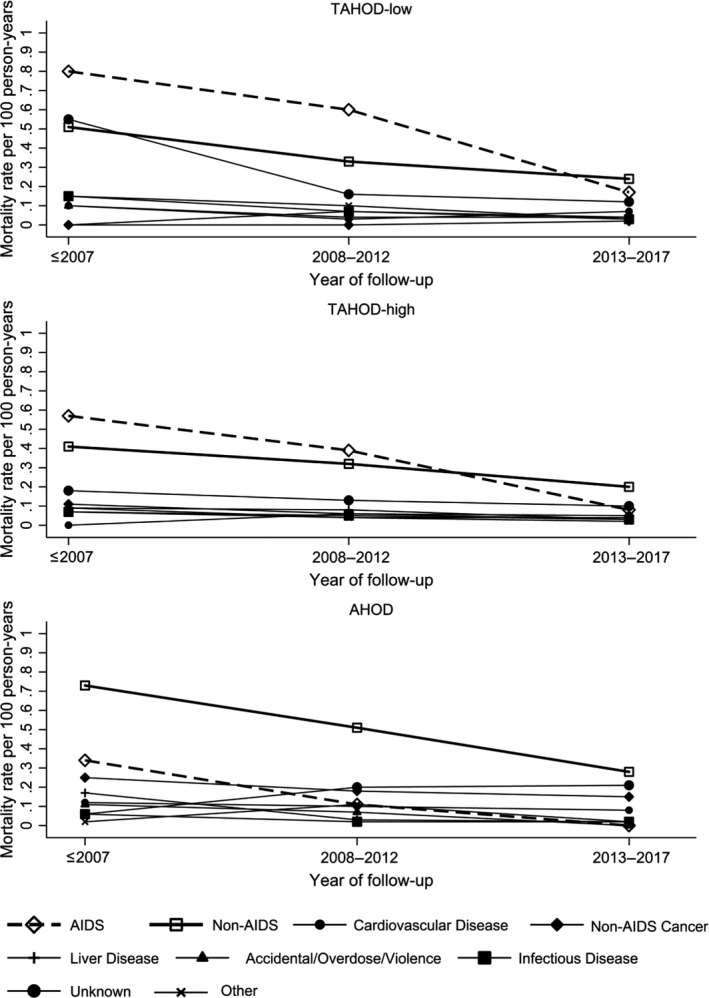
Mortality rates for different causes of death

Figure 1 demonstrates the proportions of AIDS‐related, non‐AIDS–related and unknown causes of mortality among those who died. The proportions of AIDS, non‐AIDS and unknown causes of death did not differ significantly across year groups before 2007, 2008 to 2012 and 2013 to 2017 in the TAHOD‐low cohort (*p* = 0.196). In contrast, the TAHOD‐high and AHOD cohorts demonstrated a significant difference in the proportions of AIDS, non‐AIDS and unknown causes of deaths over all year groups (TAHOD‐high: *p* = 0.025; AHOD: *p* < 0.001). Figure 2 shows a plot of mortality rates for different causes of death across calendar years of follow‐up. While AIDS mortality in TAHOD‐low and AHOD cohorts decreased with statistical significance across all year groups (TAHOD‐low: *p* = 0.001; AHOD: *p* < 0.001), the decrease across all year groups in TAHOD‐high cohort was not significant (TAHOD‐high: *p* = 0.169). Non‐AIDS mortality decreased with statistical significance across all year groups in all cohorts (TAHOD‐low: *p* = 0.010; TAHOD‐high: *p* = 0.034; AHOD: *p* = 0.027). Non‐AIDS mortality decreased at a slower rate compared to AIDS‐related deaths in TAHOD‐high (*p* = 0.039) and AHOD (*p* = 0.035) cohorts. In the TAHOD‐low cohort, the rate of decrease between the incidence of AIDS mortality and non‐AIDS mortality was not significant (*p* = 0.071). A greater decrease in the incidence of non‐AIDS mortality was shown in AHOD compared to TAHOD‐high (*p *=* *0.009) and TAHOD‐low (*p *=* *0.026) cohorts.

The incidence rate of AIDS‐related mortality during follow‐up was 0.28/100 PYS (Table 3). Univariate analysis demonstrated that mode of HIV exposure (*p *<* *0.001), HCV co‐infection (*p *=* *0.027), cohort group (*p *<* *0.001), diabetes (*p *=* *0.014), BMI, CD4, RNA and calendar year (all *p *<* *0.001) were associated with AIDS‐related mortality. In multivariate analysis adjusting for cohort groups, factors associated with AIDS‐related mortality were higher viral load (HIV RNA ≥100,000 copies/ml) compared to HIV RNA ≤400 copies/mL (aSHR = 1.95, 95% CI 1.22 to 3.13, *p *=* *0.006); and high blood glucose (aSHR = 2.56, 95% CI 1.23 to 5.33, *p *=* *0.012). Factors showing protective effects were CD4 count >200 cells/μL (aSHR = 0.04, 95% CI 0.03 to 0.07, *p *<* *0.001) compared to CD4 ≤ 100 cells/μL; and higher BMI (overweight) patients (aSHR = 0.13, 95% CI 0.03 to 0.54, *p *<* *0.005) compared to patients who were in the normal BMI range of 18.5 to 24.9. Calendar year was not statistically significant in the multivariate model; however, incidence rates of AIDS‐related deaths decreased from 0.51/100 PYS to 0.09/100 PYS in year groups from 2003 to 2017.

**Table 3:** Factors associated with AIDS mortality in the TREAT Asia HIV Observational Database and AHOD cohorts^a^

	No. of patients	Follow‐up (years)	No. of deaths	Incidence rate (/100 PYS)	Univariate		Multivariate	
					SHR (95% CI)	*p*‐value	aSHR (95% CI)	*p*‐value
Total	10385	66250	187	0.28				
Age (years)						0.116		
≤30	3011	17,628	49	0.28	1			
31 to 40	4239	27,915	70	0.25	0.98 (0.68, 1.41)	0.905		
41 to 50	2116	14,302	40	0.28	1.11 (0.73, 1.69)	0.621		
51+	1020	6405	28	0.44	1.65 (1.03, 2.62)	0.037		
Sex						0.357		
Male	7842	50,749	137	0.27	1			
Female	2544	15,501	50	0.32	1.16 (0.84 to 1.61)	0.357		
HIV mode of exposure						<0.001		
Heterosexual contact	5366	33,915	114	0.34	1			
MSM	3561	24,134	36	0.15	0.47 (0.32, 0.68)	<0.001		
Injecting drug use	764	3940	22	0.56	1.42 (0.90, 2.24)	0.134		
Blood products	93	450	3	0.67	1.55 (0.49, 4.90)	0.453		
Bisexual	140	901	4	0.44	1.31 (0.48, 3.55)	0.601		
Other/unknown	462	2908	8	0.28	0.81 (0.40, 1.66)	0.564		
CD4 (cells/μL)						<0.001		<0.001
≤100	‐	2215	102	4.60	1		1	
101 to 200	‐	4726	39	0.83	0.23 (0.16, 0.34)	<0.001	0.31 (0.21, 0.46)	<0.001
>200	‐	58,513	36	0.06	0.03 (0.02, 0.04)	<0.001	0.04 (0.03, 0.07)	<0.001
Missing	‐	795	10	1.26				
Viral load (copies/mL)						<0.001		0.019
≤400	‐	50,977	51	0.10	1		1	
401 to 10,000	‐	2469	12	0.49	3.42 (1.79, 6.52)	<0.001	1.71 (0.87, 3.34)	0.118
≥100,001	‐	4489	69	1.54	8.66 (5.84, 12.84)	<0.001	1.95 (1.22, 3.13)	0.006
Missing	‐	8314	55	0.66				
HBV co‐infection						0.6085		
Negative	7457	49,623	120	0.24	1			
Positive	705	4421	13	0.29	1.16 (0.65, 2.06)	0.608		
Missing	2224	12,206	54	0.44				
HCV co‐infection						0.027		
Negative	6892	46,347	111	0.24	1			
Positive	1110	6525	28	0.43	1.6 (1.05, 2.42)	0.027		
Missing	2384	13,377	48	0.36				
Diabetes^b^						0.014		0.012
No	‐	39,723	70	0.18	1		1	
Yes	‐	1904	8	0.42	2.52 (1.21, 5.26)	0.014	2.56 (1.23, 5.33)	0.012
Missing	‐	24,623	109	0.44				
BMI groups						<0.001		<0.001
Underweight (<18.5)	‐	4364	66	1.51	5.98 (4.24, 8.44)	<0.001	3.33 (2.31, 4.81)	<0.001
Normal range (18.5 to 24.9)	‐	31,289	65	0.21	1		1	
Overweight (≥25)	‐	12,168	2	0.02	0.09 (0.02, 0.37)	0.001	0.13 (0.03, 0.54)	0.005
Missing	‐	18,429	54	0.29				
Cohort groups						<0.001		0.834
TAHOD‐low	3240	17,833	72	0.40	1		1	
TAHOD‐high	4429	29,242	86	0.29	0.82 (0.60, 1.13)	0.228	0.99 (0.68, 1.44)	0.953
AHOD	2717	19,175	29	0.15	0.44 (0.29, 0.68)	<0.001	1.15 (0.64, 2.07)	0.629
Calendar year						<0.001		
≤2002	‐	2046	8	0.39	0.58 (0.28, 1.21)	0.148		
2003 to 2007	‐	11,006	56	0.51	1			
2008 to 2012	‐	25,968	98	0.38	0.87 (0.62, 1.21)	0.414		
2013 to 2017	‐	27,230	25	0.09	0.34 (0.21, 0.54)	<0.001		

Global *p*‐values were calculated by excluding the missing category. AHOD, Australian HIV Observational Database; aSHR, adjusted sub‐hazard ratio; BMI, body mass index; CI, confidence interval; HBV, hepatitis B virus; HCV, hepatitis C virus; MSM, Men who have sex with men; PYS, person‐years; SHR, sub‐hazard ratio; TAHOD, TREAT Asia HIV Observational Database.

^a^Cohorts were grouped as AHOD (all high‐income sites), TAHOD‐high (high/upper‐middle income countries) and TAHOD‐low (low‐middle/low income countries). TAHOD sites were split into high/upper‐middle income and low‐middle/low income settings based on World Bank classifications. Time‐fixed covariates: Age, Sex, HIV mode of exposure, HBV co‐infection, HCV co‐infection, Cohort Groups; time‐updated covariates: CD4, Viral Load, Diabetes, BMI Groups and Calendar year. ^b^Diabetes was defined as documentation of one fasting blood glucose measurement ≥7 mmol/L.

The incidence rate of non‐AIDs–related mortality during follow‐up was 0.51 per 100 PYS (Table 4). Factors associated with non‐AIDS mortality in the multivariate analysis were: older age (31 to 40 years: aSHR = 1.40, 95% CI 1.02 to 1.93, *p *=* *0.039; 41 to 50 years: aSHR = 1.58, 95% CI 1.10 to 2.27, *p *=* *0.013; and >50 years: aSHR = 4.43, 95% CI 3.15 to 6.25, *p *<* *0.001) compared to age ≤30 years; HBV co‐infection (aSHR = 1.87, 95% CI 1.32 to 2.65, *p *<* *0.001); HCV co‐infection (aSHR = 1.96, 95% CI 1.44 to 2.67, *p *<* *0.001); high blood glucose (aSHR = 1.77, 95% CI 1.09 to 2.86, *p *=* *0.021); and AHOD cohort (aSHR = 1.72, 95% CI 1.20 to 2.46, *p *=* *0.003) compared to TAHOD‐low. Non‐AIDS mortality was less likely in females (aSHR = 0.5, 95% CI 0.35 to 0.70, *p *<* *0.001); higher BMI (overweight: aSHR = 0.69, 95% CI 0.47 to 1.01, *p *<* *0.054) compared to normal weight range (BMI of 18.5 to 24.9); and later calendar years of follow‐up (2008 to 2012: aSHR = 0.72, 95% CI 0.54 to 0.96, *p *=* *0.027; 2013 to 2017: aSHR = 0.64, 95% CI 0.47 to 0.87, *p *=* *0.004) compared to year group 2003 to 2007. Higher CD4 count (101 to 200 cells/μL: aSHR = 0.61, 95% CI 0.42 to 0.90, *p *=* *0.011; >200 cells/μL: aSHR = 0.23, 95% CI 0.17 to 0.33, *p *<* *0.001) compared to CD4 ≤ 100 cells/μL was also associated with lower non‐AIDS mortality. However, since the multivariable models were adjusted for time‐updated CD4 count and viral load, changes over time in the rate of non‐AIDS mortality are not entirely explained by CD4 count and viral load.

**Table 4:** Factors associated with non‐AIDS mortality in the TREAT Asia HIV Observational Database and AHOD^a^

	No. patients	Follow‐up (years)	No. of deaths	Incidence rate (/100 PYS)	Univariate		Multivariate	
					SHR (95% CI)	*p*‐value	aSHR (95% CI)	*p*‐value
Total	10,385	66,250	335	0.51				
Age (years)						<0.001		<0.001
≤30	3011	17,628	59	0.33	1		1	
31 to 40	4239	27,915	117	0.42	1.31 (0.96, 1.79)	0.091	1.40 (1.02, 1.93)	0.039
41 to 50	2116	14,302	68	0.48	1.51 (1.06, 2.14)	0.021	1.58 (1.10, 2.27)	0.013
51+	1020	6405	91	1.42	4.39 (3.17, 6.08)	<0.001	4.43 (3.15, 6.25)	<0.001
Sex						<0.001		<0.001
Male	7842	50,749	299	0.59	1		1	
Female	2544	15,501	36	0.23	0.40 (0.28, 0.57)	<0.001	0.50 (0.35, 0.70)	<0.001
HIV mode of exposure						0.005		
Heterosexual contact	5366	33,915	150	0.44	1			
MSM	3561	24,134	121	0.50	1.13 (0.89, 1.44)	0.298		
Injecting drug use	764	3940	39	0.99	1.99 (1.40, 2.83)	<0.001		
Blood products	93	450	5	1.11	1.98 (0.82, 4.83)	0.131		
Bisexual	140	901	6	0.67	1.52 (0.67, 3.46)	0.313		
Other/unknown	462	2908	14	0.48	1.06 (0.61, 1.84)	0.826		
CD4 (cells/μL)						<0.001		<0.001
≤100	‐	2215	63	2.84	1		1	
101 to 200	‐	4726	57	1.21	0.57 (0.40, 0.83)	0.003	0.61 (0.42, 0.90)	0.011
>200	‐	58,513	204	0.35	0.20 (0.15, 0.26)	<0.001	0.23 (0.17, 0.33)	<0.001
Missing	‐	795	11	1.38				
Viral load (copies/mL)						<0.001		
≤400	‐	50,977	204	0.40	1			
401 to 10,000	‐	2469	15	0.61	1.31 (0.77, 2.22)	0.313		
≥100,001	‐	4489	57	1.27	2.42 (1.78, 3.29)	<0.001		
Missing	‐	8314	59	0.71				
HBV co‐infection						<0.001		<0.001
Negative	7457	49,623	230	0.46	1		1	
Positive	705	4421	37	0.84	1.80 (1.27, 2.54)	0.001	1.87 (1.32, 2.65)	<0.001
Missing	2224	12,206	68	0.56				
HCV co‐infection						<0.001		<0.001
Negative	6892	46,347	200	0.43	1		1	
Positive	1110	6525	63	0.97	2.08 (1.57, 2.76)	<0.001	1.96 (1.44, 2.67)	<0.001
Missing	2384	13,377	72	0.54				
Diabetes^b^						<0.001		0.021
No	‐	39,723	164	0.41	1		1	
Yes	‐	1904	19	1	2.44 (1.51, 3.92)	<0.001	1.77 (1.09, 2.86)	0.021
Missing	‐	24,623	152	0.62				
BMI groups						<0.001		<0.001
Underweight (<18.5)	‐	4364	56	1.28	2.69 (1.96, 3.69)	<0.001	2.21 (1.56, 3.12)	<0.001
Normal range (18.5 to 24.9)	‐	31,289	127	0.41	1		1	
Overweight (≥25)	‐	12,168	36	0.30	0.75 (0.52, 1.09)	0.135	0.69 (0.47, 1.01)	0.054
Missing	‐	18,429	116	0.63				
Cohort groups						0.003		0.001
TAHOD‐low	3240	17,833	87	0.49	1		1	
TAHOD‐high	4429	29,242	122	0.42	0.89 (0.68, 1.18)	0.421	0.99 (0.73, 1.34)	0.936
AHOD	2717	19,175	126	0.66	1.35 (1.03, 1.78)	0.029	1.72 (1.20, 2.46)	0.003
Calendar year						<0.001		0.028
≤2002	‐	2046	13	0.64	0.77 (0.43, 1.39)	0.39	0.66 (0.36, 1.23)	0.194
2003 to 2007	‐	11,006	86	0.78	1		1	
2008 to 2012	‐	25,968	136	0.52	0.68 (0.52, 0.89)	0.006	0.72 (0.54, 0.96)	0.027
2013 to 2017	‐	27,230	100	0.37	0.50 (0.37, 0.67)	<0.001	0.64 (0.47, 0.87)	0.004

Global *p*‐values were calculated by excluding the missing category. AHOD, Australian HIV Observational Database; aSHR, adjusted sub‐hazard ratio; BMI, body mass index; CI, confidence interval; HBV, hepatitis B virus; HCV, hepatitis C virus; MSM, Men who have sex with men; No., number; PYS, person‐years; SHR, sub‐hazard ratio; TAHOD, TREAT Asia HIV Observational Database.

^a^Cohorts were grouped as AHOD (all high‐income sites), TAHOD‐high (high/upper‐middle income countries) and TAHOD‐low (low‐middle/low income countries). TAHOD sites were split into high/upper‐middle income and low‐middle/low income settings based on World Bank classifications. Time‐fixed covariates: age, sex, HIV mode of exposure, HBV co‐infection, HCV co‐infection, cohort groups; time‐updated covariates: CD4, viral load, diabetes, BMI groups and calendar year. ^b^Diabetes was defined as documentation of one fasting blood glucose measurement ≥7 mmol/L.

Tables S1 and S2 show that by including time‐updated age, sex and CD4 in the final multivariate model, there was a similar increasing trend in the hazard for mortality with increasing age, which has now shown significant associations with both AIDS and non‐AIDS mortality. Other risk factors and their effects are similar to those observed in the main analyses.

## 4 Discussion

In our cross‐regional observational cohorts who received cART, the overall incidence rate of mortality over a median follow‐up time of 6.0 years was 0.28/100 PYS from AIDS‐related and 0.51/100 PYS from non‐AIDS–related causes. Moreover, incidence rates for AIDS‐related and non‐AIDS–related mortality have decreased over the years with the AHOD cohort showing higher hazard for non‐AIDS deaths compared to TAHOD‐low sites.

With the introduction of cART, AIDS‐related death has decreased followed by an increase in non‐AIDS death 4. According to the D:A:D multicohort collaboration data, the mortality rate of all‐cause death was 17.5/1000 PYS in 1999 to 2000 and decreased to 9.1/1000 PYS in 2009 to 2011 1. AIDS‐related deaths demonstrated similar decreases in death rates over the same period from 5.9/1000 PYS to 2.0/1000 PYS 1. In our study, incidence rates of AIDS‐related deaths decreased from 0.51/100 PYS to 0.09/100 PYS from year groups 2003 to 2007 to 2013 to 2017. AIDS‐related COD was the most common COD in the TAHOD cohort in year group ≤2007, and over the years there was a decrease in the overall incidence of AIDS deaths for all cohorts. These findings are thought to be associated with the introduction of effective cART in resource‐limited settings, which slows the decline of CD4 cell counts, extending the time to AIDS‐related death and the total number of AIDS deaths itself 3, 16.

In a Swiss HIV cohort study, AIDS‐related death was highest in 1992 (11.0/100 PYS, 95% CI 9.94 to 12.1) and decreased to 0.211/100 PYS (95% CI 0.122 to 0.363) in 2010 6. Non‐AIDS–related mortality decreased from 1.74/100 PYS (95% CI 1.36 to 2.23) in 1993 to 0.86/100 PYS (95% CI 0.657 to 1.13) in 2010 6. The results of our study also demonstrated a declining trend in non‐AIDS deaths in the later calendar years. Additionally, we found that there has been an increase in the proportion of unknown deaths recorded in the AHOD cohort. This increase in unknown deaths in AHOD was due to delay in reporting of the causes of death by the site. Mortality rates for AIDS and non‐AIDS causes of death have declined over the years, with the incidence rate of non‐AIDS–related death remaining high in AHOD, but declining, compared to other causes of death. Overall, there are higher incidences of non‐AIDS deaths than AIDS‐related mortality in later years in the Asia‐Pacific region, similar to those demonstrated in other regions 1, 3.

HIV‐infected patients are prone to increased risk of cancer, and an estimated 30% to 40% of patients will develop cancer during their lifetime 17. According to the D:A:D multicohort collaboration data, the incidence of non‐AIDS cancers increased from 1.6/1000 PYS (1999 to 2000) to 2.1/1000 PYS (2009 to 2011) 1. As people with HIV have a longer lifespan with potent cART, more patients develop malignancies including cancers not associated with AIDS 18, 19, and this might also explain the higher proportion of non‐AIDS deaths found in our cohorts. However, in this study, mortality rates for non‐AIDS–related malignancies decreased from 0.16/100 PYS in year groups before 2007 to 0.07/100 PYS in year groups 2013 to 2017. Early treatment due to aggressive screening may be accountable for the decrease in non‐AIDS malignancy mortality. Moreover, more detection of cancer due to aggressive screening in the AHOD cohort may be the reason for the higher risk of non‐AIDS–related deaths in the AHOD cohort. The number of cardiovascular–related deaths was also higher in the AHOD cohort. Although there were no differences between cohorts for AIDS‐related mortality, the AHOD cohort was at higher risk of non‐AIDS mortality compared to both TAHOD cohorts with almost twice the hazard of non‐AIDS mortality compared to TAHOD‐low. The non‐AIDS mortality highlights the importance for healthcare professionals to provide more comprehensive healthcare that includes screening for malignancy or cardiovascular disease.

Cardiovascular events associated with lower CD4 cell counts were suggested in both the Strategies for Management of Antiretroviral Therapy (SMART) and D:A:D studies 20, 21. Furthermore, immunodeficiency, identified by lower CD4 cell counts, was also strongly associated with liver‐related non‐AIDS death 22. In a prospective cohort study of HIV‐infected patients diagnosed at the age of 50 years or more, the baseline CD4 cell count was lower and presented shorter survival 23. Moreover, continuing improvements in CD4 cell count over time were attributable to the reduction in AIDS‐related deaths 1. Our findings are similar to results of these studies, demonstrating lower CD4 cell counts and older age as independent risk factors for both AIDS and non‐AIDS mortality. Our study was also comparable to studies conducted throughout South America and the Caribbean, which have shown that low BMI and low haemoglobin were predictive of mortality, especially in the early phases of cART initiation 24. Moreover, one prospective cohort study which followed up 1119 HIV patients for a median number of 8.2 years demonstrated that HIV‐infected persons with food insecurity, categorized by low BMI, were about two times more likely to experience mortality 25. In our study, low BMI categorized as underweight was also a risk factor for AIDS and non‐AIDS mortality, putting emphasis on the need to incorporate nutritional support in HIV treatment programs.

There are several limitations to our study. Information on patient adherence data is not collected in AHOD and it is possible that poor patient compliance or adherence may have been a reason for AIDS death. The delay in reporting in the causes of death in AHOD may lead to incomplete COD ascertainment and underestimation of the AIDS and non‐AIDS causes of death. However, the majority of the unknown causes of death would be traced at a later date. While TAHOD represents a primarily urban referral patient population, crossing from low to high country income categories, it is less representative of national‐level data. There is also the risk of unascertained mortality among those with LTFU.

## 5 Conclusions

We observed that the rates of AIDS‐related and non‐AIDS‐related mortality in our Asia‐Pacific cohorts have decreased over the past years. Factors associated with increased risk of AIDS‐related mortality were higher viral load, high blood glucose, low CD4 cell count and low BMI. Older age, hepatitis B and C co‐infection, high blood glucose, low CD4 cell count and BMI were associated with increased non‐AIDS death. Blood glucose levels, CD4 cell count and being underweight were risk factors for both categories of deaths. The higher risk for non‐AIDS–associated deaths in the AHOD cohort compared to the lower‐middle income setting in TAHOD may be related to older age and higher per cent of reporting number of deaths. The slower rate in the decline of non‐AIDS deaths and the higher proportions of non‐AIDS deaths still persisting over AIDS‐related deaths demonstrate that longer lifespans contribute to increased risk of developing cancers and other conditions more common with older age, resulting in more deaths from non‐AIDS causes.

## Competing interests

None of the authors have competing interests to declare.

## Authors’ contributions

JYC contributed to conceptualization and design of the study; IYJ wrote the first draft of the manuscript and interpreted the results; DR contributed to acquisition of data, data analysis and interpretation; IW, CO, MG and IA contributed to editing of the article; all authors have read and approved the final manuscript.

## Supporting information


**Table S1.** Factors associated with AIDS mortality in the TREAT Asia HIV Observational Database and Australian HIV Observational Database cohorts with age as a time‐dependent covariate including age, sex and CD4 in the multivariate model^a^
**Table S2.** Factors associated with non‐AIDS mortality in the TREAT Asia HIV Observational Database and Australian HIV Observational Database cohorts with age as a time‐dependent covariate including age, sex and CD4 in the multivariate model^a^
