# Supplementary material for: Trends in mortality among ART‐treated HIV‐infected adults in the Asia‐Pacific region between 1999 and 2017: results from the TREAT Asia HIV Observational Database (TAHOD) and Australian HIV Observational Database (AHOD) of IeDEA Asia‐Pacific
Source: J Int AIDS Soc. 2019 Jan 7;22(1):e25219. doi: 10.1002/jia2.25219 (PMC6322485; doi:10.1002/jia2.25219)
Supplement: Supplementary file 1 — Table S1. Factors associated with AIDS mortality in the TREAT Asia HIV Observational Database and Australian HIV Observational Database cohorts with age as a time‐dependent covariate including age, sex and CD4 in the multivariate modela Table S2. Factors associated with non‐AIDS mortality in the TREAT Asia HIV Observational Database and Australian HIV Observational Database cohorts with age as a time‐dependent covariate including age, sex and CD4 in the multivariate modela [file JIA2-22-e25219-s001.docx]

**Supplementary table 1: Factors associated with AIDS mortality in the TREAT Asia HIV Observational Database and Australian HIV Observational Database cohorts with age as a time-dependant covariate including age, sex and CD4 in the multivariate model§.**

|  |  |  |  |  | **Univariate** | | | **Multivariate** | | |
| --- | --- | --- | --- | --- | --- | --- | --- | --- | --- | --- |
|  | **No patients** | **Follow up (years)** | **No of deaths** | **Incidence rate (/100pys)** | **SHR** | **95% CI** | **p-value** | **aSHR** | **95% CI** | **p-value** |
| **Total** | 10385 | 66250 | 187 | 0.28 |  |  |  |  |  |  |
| **Age (years)** |  |  |  |  |  |  | 0.519 |  |  | **0.001** |
| ≤ 30 | ~ | 6161 | 25 | 0.41 | 1 |  |  | 1 |  |  |
| 31-40 | ~ | 23184 | 71 | 0.31 | 1.15 | (0.73, 1.81) | 1 | 1.54 | (0.97, 2.44) | 0.065 |
| 41-50 | ~ | 22184 | 54 | 0.24 | 1.22 | (0.76, 1.96) | 0.404 | 2.00 | (1.24, 3.24) | 0.005 |
| 51+ | ~ | 14721 | 37 | 0.25 | 1.47 | (0.87, 2.50) | 0.151 | 2.81 | (1.63, 4.86) | <0.001 |
|  |  |  |  |  |  |  |  |  |  |  |
| **Sex** |  |  |  |  |  |  | 0.357 |  |  | 0.653 |
| Male | 7842 | 50749 | 137 | 0.27 | 1 |  |  | 1 |  |  |
| Female | 2544 | 15501 | 50 | 0.32 | 1.16 | (0.84 - 1.61) | 0.357 | 1.08 | (0.77, 1.52) | 0.653 |
|  |  |  |  |  |  |  |  |  |  |  |
| **HIV mode of exposure** |  |  |  |  |  |  | < 0.001 |  |  |  |
| Heterosexual contact | 5366 | 33915 | 114 | 0.34 | 1 |  |  |  |  |  |
| MSM | 3561 | 24134 | 36 | 0.15 | 0.47 | (0.32, 0.68) | <0.001 |  |  |  |
| Injecting drug use | 764 | 3940 | 22 | 0.56 | 1.42 | (0.90, 2.24) | 0.134 |  |  |  |
| Blood products | 93 | 450 | 3 | 0.67 | 1.55 | (0.49, 4.90) | 0.453 |  |  |  |
| Bisexual | 140 | 901 | 4 | 0.44 | 1.31 | (0.48, 3.55) | 0.601 |  |  |  |
| Other/Unknown | 462 | 2908 | 8 | 0.28 | 0.81 | (0.40, 1.66) | 0.564 |  |  |  |
|  |  |  |  |  |  |  |  |  |  |  |
| **CD4 (cells/µL)** |  |  |  |  |  |  | < 0.001 |  |  | **0.007** |
| ≤ 100 | ~ | 2215 | 102 | 4.6 | 1 |  |  | 1 |  |  |
| 101-200 | ~ | 4726 | 39 | 0.83 | 0.23 | (0.16, 0.34) | <0.001 | 0.31 | (0.21, 0.46) | <0.001 |
| >200 | ~ | 58513 | 36 | 0.06 | 0.03 | (0.02, 0.04) | <0.001 | 0.04 | (0.03, 0.07) | <0.001 |
| Missing | ~ | 795 | 10 | 1.26 |  |  |  |  |  |  |
|  |  |  |  |  |  |  |  |  |  |  |
| **Viral Load (copies/mL)** |  |  |  |  |  |  | < 0.001 |  |  | **< 0.001** |
| ≤ 400 | ~ | 50977 | 51 | 0.1 | 1 |  |  | 1 |  |  |
| 401-10000 | ~ | 2469 | 12 | 0.49 | 3.42 | (1.79, 6.52) | <0.001 | 1.81 | (0.92, 3.56) | 0.086 |
| ≥ 100001 | ~ | 4489 | 69 | 1.54 | 8.66 | (5.84, 12.84) | <0.001 | 2.12 | (1.32, 3.42) | 0.002 |
| Missing | ~ | 8314 | 55 | 0.66 |  |  |  |  |  |  |
|  |  |  |  |  |  |  |  |  |  |  |
| **HBV co-infection** |  |  |  |  |  |  | 0.609 |  |  |  |
| Negative | 7457 | 49623 | 120 | 0.24 | 1 |  |  |  |  |  |
| Positive | 705 | 4421 | 13 | 0.29 | 1.16 | (0.65, 2.06) | 0.608 |  |  |  |
| Missing | 2224 | 12206 | 54 | 0.44 |  |  |  |  |  |  |
|  |  |  |  |  |  |  |  |  |  |  |
| **HCV co-infection** |  |  |  |  |  |  | 0.027 |  |  |  |
| Negative | 6892 | 46347 | 111 | 0.24 | 1 |  |  |  |  |  |
| Positive | 1110 | 6525 | 28 | 0.43 | 1.60 | (1.05, 2.42) | 0.027 |  |  |  |
| Missing | 2384 | 13377 | 48 | 0.36 |  |  |  |  |  |  |
|  |  |  |  |  |  |  |  |  |  |  |
| **Diabetes*** |  |  |  |  |  |  | 0.014 |  |  | **0.042** |
| No | ~ | 39723 | 70 | 0.18 | 1 |  |  | 1 |  |  |
| Yes | ~ | 1904 | 8 | 0.42 | 2.52 | (1.21, 5.26) | 0.014 | 2.20 | (1.03, 4.70) | 0.042 |
| Missing | ~ | 24623 | 109 | 0.44 |  |  |  |  |  |  |
|  |  |  |  |  |  |  |  |  |  |  |
| **BMI Groups** |  |  |  |  |  |  | < 0.001 |  |  | **< 0.001** |
| Underweight (<18.5) | ~ | 4364 | 66 | 1.51 | 5.98 | (4.24, 8.44) | <0.001 | 3.44 | (2.37, 4.98) | <0.001 |
| Normal Range (18.5 - 24.9) | ~ | 31289 | 65 | 0.21 | 1 |  |  | 1 |  |  |
| Overweight(≥ 25) | ~ | 12168 | 2 | 0.02 | 0.09 | (0.02, 0.37) | 0.001 | 0.13 | (0.03, 0.52) | 0.004 |
| Missing | ~ | 18429 | 54 | 0.29 |  |  |  |  |  |  |
|  |  |  |  |  |  |  |  |  |  |  |
| **Cohort Groups** |  |  |  |  |  |  | < 0.001 |  |  | 0.849 |
| TAHOD-low | 3240 | 17833 | 72 | 0.40 | 1 |  |  | 1 |  |  |
| TAHOD-high | 4429 | 29242 | 86 | 0.29 | 0.82 | (0.60, 1.13) | 0.228 | 0.90 | (0.62, 1.32) | 0.606 |
| AHOD | 2717 | 19175 | 29 | 0.15 | 0.44 | (0.29, 0.68) | <0.001 | 0.99 | (0.56, 1.76) | 0.977 |
|  |  |  |  |  |  |  |  |  |  |  |
| **Calendar year** |  |  |  |  |  |  | < 0.001 |  |  |  |
| ≤ 2002 | ~ | 2046 | 8 | 0.39 | 0.58 | (0.28, 1.21) | 0.148 |  |  |  |
| 2003-2007 | ~ | 11006 | 56 | 0.51 | 1 |  |  |  |  |  |
| 2008-2012 | ~ | 25968 | 98 | 0.38 | 0.87 | (0.62, 1.21) | 0.414 |  |  |  |
| 2013-2017 | ~ | 27230 | 25 | 0.09 | 0.34 | (0.21, 0.54) | <0.001 |  |  |  |

TAHOD, TREAT Asia HIV Observational Database; AHOD, Australian HIV Observational Database; pys, person years; No., number; SHR, sub-hazard ratio; aSHR- adjusted sub-hazard ratio; MSM, Men who have sex with men; HBV, hepatitis B virus; HCV, hepatitis C virus; BMI, body mass index

§Cohorts were grouped as AHOD (all high-income sites), TAHOD-high (high/upper-middle income countries) and TAHOD-low (low-middle/low income countries). TAHOD sites were split into high/upper-middle income and low-middle/low income settings based on World Bank classifications. Time-fixed covariates: Sex, HIV mode of exposure, HBV co-infection, HCV co-infection, Cohort Groups; time-updated covariates: Age, CD4, Viral Load, Diabetes, BMI Groups and Calendar year.

*Diabetes was defined as documentation of one fasting blood glucose measurement ≥ 7 mmol/L.

Global p-values were calculated by excluding the missing category.

**Supplementary table 2. Factors associated with non-AIDS mortality in the TREAT Asia HIV Observational Database and Australian HIV Observational Database cohorts with age as a time-dependant covariate including age, sex and CD4 in the multivariate model§.**

|  |  |  |  |  | **Univariate** | | | **Multivariate** | | |
| --- | --- | --- | --- | --- | --- | --- | --- | --- | --- | --- |
|  | **No patients** | **Follow up (years)** | **No of deaths** | **Incidence rate (/100pys)** | **SHR** | **95% CI** | **p-value** | **aSHR** | **95% CI** | **p-value** |
| **Total** | 10385 | 66250 | 335 | 0.51 |  |  |  |  |  |  |
| **Age (years)** |  |  |  |  |  |  | <0.001 |  |  | **<0.001** |
| ≤ 30 | 3011 | 17628 | 59 | 0.33 | 1 |  |  | 1 |  |  |
| 31-40 | 4239 | 27915 | 117 | 0.42 | 1.36 | (0.85, 2.19) | 0.199 | 1.60 | (0.99, 2.60) | 0.058 |
| 41-50 | 2116 | 14302 | 68 | 0.48 | 1.69 | (1.05, 2.71) | 0.030 | 2.13 | (1.31, 3.48) | 0.002 |
| 51+ | 1020 | 6405 | 91 | 1.42 | 3.95 | (2.49, 6.26) | <0.001 | 5.03 | (3.10, 8.14) | <0.001 |
|  |  |  |  |  |  |  |  |  |  |  |
| **Sex** |  |  |  |  |  |  | <0.001 |  |  | **<0.001** |
| Male | 7842 | 50749 | 299 | 0.59 | 1 |  |  | 1 |  |  |
| Female | 2544 | 15501 | 36 | 0.23 | 0.40 | (0.28, 0.57) | <0.001 | 0.49 | (0.35, 0.70) | <0.001 |
|  |  |  |  |  |  |  |  |  |  |  |
| **HIV mode of exposure** |  |  |  |  |  |  | 0.005 |  |  |  |
| Heterosexual contact | 5366 | 33915 | 150 | 0.44 | 1 |  |  |  |  |  |
| MSM | 3561 | 24134 | 121 | 0.50 | 1.13 | (0.89, 1.44) | 0.298 |  |  |  |
| Injecting drug use | 764 | 3940 | 39 | 0.99 | 1.99 | (1.40, 2.83) | <0.001 |  |  |  |
| Blood products | 93 | 450 | 5 | 1.11 | 1.98 | (0.82, 4.83) | 0.131 |  |  |  |
| Bisexual | 140 | 901 | 6 | 0.67 | 1.52 | (0.67, 3.46) | 0.313 |  |  |  |
| Other/Unknown | 462 | 2908 | 14 | 0.48 | 1.06 | (0.61, 1.84) | 0.826 |  |  |  |
|  |  |  |  |  |  |  |  |  |  |  |
| **CD4 (cells/µL)** |  |  |  |  |  |  | <0.001 |  |  | **<0.001** |
| ≤ 100 | ~ | 2215 | 63 | 2.84 | 1 |  |  | 1 |  |  |
| 101-200 | ~ | 4726 | 57 | 1.21 | 0.57 | (0.40, 0.83) | 0.003 | 0.59 | (0.41, 0.86) | 0.006 |
| >200 | ~ | 58513 | 204 | 0.35 | 0.20 | (0.15, 0.26) | <0.001 | 0.21 | (0.15, 0.30) | <0.001 |
| Missing | ~ | 795 | 11 | 1.38 |  |  |  |  |  |  |
|  |  |  |  |  |  |  |  |  |  |  |
| **Viral Load (copies/mL)** |  |  |  |  |  |  | <0.001 |  |  |  |
| ≤ 400 | ~ | 50977 | 204 | 0.40 | 1 |  |  |  |  |  |
| 401-10000 | ~ | 2469 | 15 | 0.61 | 1.31 | (0.77, 2.22) | 0.313 |  |  |  |
| ≥ 100001 | ~ | 4489 | 57 | 1.27 | 2.42 | (1.78, 3.29) | <0.001 |  |  |  |
| Missing | ~ | 8314 | 59 | 0.71 |  |  |  |  |  |  |
|  |  |  |  |  |  |  |  |  |  |  |
| **Hepatitis B co-infection** |  |  |  |  |  |  | <0.001 |  |  | **<0.001** |
| Negative | 7457 | 49623 | 230 | 0.46 | 1 |  |  | 1 |  |  |
| Positive | 705 | 4421 | 37 | 0.84 | 1.80 | (1.27, 2.54) | 0.001 | 1.83 | (1.29, 2.60) | 0.001 |
| Missing | 2224 | 12206 | 68 | 0.56 |  |  |  |  |  |  |
|  |  |  |  |  |  |  |  |  |  |  |
| **Hepatitis C co-infection** |  |  |  |  |  |  | <0.001 |  |  | **<0.001** |
| Negative | 6,892 | 46347 | 200 | 0.43 | 1 |  |  | 1 |  |  |
| Positive | 1110 | 6525 | 63 | 0.97 | 2.08 | (1.57, 2.76) | <0.001 | 1.94 | (1.43, 2.63) | <0.001 |
| Missing | 2384 | 13377 | 72 | 0.54 |  |  |  |  |  |  |
|  |  |  |  |  |  |  |  |  |  |  |
| **Diabetes*** |  |  |  |  |  |  | <0.001 |  |  | **0.024** |
| No | ~ | 39723 | 164 | 0.41 | 1 |  |  | 1 |  |  |
| Yes | ~ | 1904 | 19 | 1 | 2.44 | (1.51, 3.92) | <0.001 | 1.75 | (1.08, 2.86) | 0.024 |
| Missing | ~ | 24623 | 152 | 0.62 |  |  |  |  |  |  |
|  |  |  |  |  |  |  |  |  |  |  |
| **BMI Groups** |  |  |  |  |  |  | <0.001 |  |  | **<0.001** |
| Underweight (<18.5) | ~ | 4364 | 56 | 1.28 | 2.69 | (1.96, 3.69) | <0.001 | 2.27 | (1.61, 3.21) | <0.001 |
| Normal Range (18.5 - 24.9) | ~ | 31289 | 127 | 0.41 | 1 |  |  | 1 |  |  |
| Overweight(≥ 25) | ~ | 12168 | 36 | 0.30 | 0.75 | (0.52, 1.09) | 0.135 | 0.67 | (0.46, 0.99) | 0.042 |
| Missing | ~ | 18429 | 116 | 0.63 |  |  |  |  |  |  |
|  |  |  |  |  |  |  |  |  |  |  |
| **Cohort Groups** |  |  |  |  |  |  | 0.003 |  |  | **0.007** |
| TAHOD-low | 3240 | 17833 | 87 | 0.49 | 1 |  |  | 1 |  |  |
| TAHOD-high | 4429 | 29242 | 122 | 0.42 | 0.89 | (0.68, 1.18) | 0.421 | 0.97 | (0.72, 1.30) | 0.820 |
| AHOD | 2717 | 19175 | 126 | 0.66 | 1.35 | (1.03, 1.78) | 0.029 | 1.57 | (1.10, 2.24) | 0.014 |
|  |  |  |  |  |  |  |  |  |  |  |
| **Calendar year** |  |  |  |  |  |  | <0.001 |  |  | **0.025** |
| ≤ 2002 | ~ | 2046 | 13 | 0.64 | 0.77 | (0.43, 1.39) | 0.390 | 0.70 | (0.38, 1.29) | 0.254 |
| 2003-2007 | ~ | 11006 | 86 | 0.78 | 1 |  |  | 1 |  |  |
| 2008-2012 | ~ | 25968 | 136 | 0.52 | 0.68 | (0.52, 0.89) | 0.006 | 0.73 | (0.55, 0.98) | 0.034 |
| 2013-2017 | ~ | 27230 | 100 | 0.37 | 0.50 | (0.37, 0.67) | <0.001 | 0.62 | (0.46, 0.85) | 0.003 |

TAHOD, TREAT Asia HIV Observational Database; AHOD, Australian HIV Observational Database; pys, person years; No., number; SHR, sub-hazard ratio; aSHR- adjusted sub-hazard ratio; MSM, Men who have sex with men; HBV, hepatitis B virus; HCV, hepatitis C virus; BMI, body mass index

§Cohorts were grouped as AHOD (all high-income sites), TAHOD-high (high/upper-middle income countries) and TAHOD-low (low-middle/low income countries). TAHOD sites were split into high/upper-middle income and low-middle/low income settings based on World Bank classifications. Time-fixed covariates: Sex, HIV mode of exposure, HBV co-infection, HCV co-infection, Cohort Groups; time-updated covariates: Age, CD4, Viral Load, Diabetes, BMI Groups and Calendar year.

*Diabetes was defined as documentation of one fasting blood glucose measurement ≥ 7 mmol/L.

Global p-values were calculated by excluding the missing category.
